# Cardiac Magnetic Resonance for Structural Aortic Valve Stenosis Procedures

**DOI:** 10.3390/jcm13175184

**Published:** 2024-09-01

**Authors:** Marcos Ferrández-Escarabajal, Michael Hadley, Javier Sanz

**Affiliations:** The Zena and Michael A. Wiener Cardiovascular Institute, Icahn School of Medicine at Mount Sinai, New York, NY 10029, USA; marcos.ferrandezescarabajal@mountsinai.org (M.F.-E.); michael.hadley@mountsinai.org (M.H.)

**Keywords:** cardiac magnetic resonance, transcatheter aortic valve replacement, aortic stenosis, aortic regurgitation, myocardium

## Abstract

The number of structural aortic valve procedures has increased significantly in recent years. Pre-procedural planning and follow-up with noninvasive testing are essential. Although cardiac magnetic resonance (CMR) is the gold standard for assessing left ventricular mass, volume, and function, it is not performed routinely in patients undergoing structural interventions. CMR can provide useful information for pre- and post-procedural assessment, including quantification of cardiac function, myocardial assessment, grading of the severity of valvular heart disease, and evaluation of extracardiac anatomy while avoiding the limitations of other non-invasive modalities. Here, we review the use cases, future perspectives, and limitations of CMR for patients undergoing structural aortic valve procedures.

## 1. Introduction

Aortic stenosis (AS) is an increasingly common valvular disease due to the progressive aging of the population. Transcatheter aortic valve replacement (TAVR) provides a minimally invasive treatment. The 2021 European Society of Cardiology and European Association for Cardio-Thoracic Surgery Guidelines for the management of valvular heart disease recommend TAVR in patients ≥75 years or in those who have high surgical risk or are unsuitable for surgery [1]. The positive results of TAVR also in intermediate-risk and low-risk patients suggest that this procedure will become increasingly common in the coming years [2,3]. Although the development of TAVR represents a radical scientific advance, it also presents several challenges for pre- and post-procedural assessment.

Traditionally, transthoracic echocardiography (TTE) and cardiac computed tomography (CCT) guide the diagnosis and procedural planning of patients with severe AS. However, these tests have limitations (see Table 1 and throughout the text below). With cardiac magnetic resonance (CMR), it is now possible, in a single study, to evaluate the severity of AS, annulus measurements, vascular access, and cardiac volumes and function. Additionally, CMR constitutes the current noninvasive gold standard for myocardial characterization. A myocardial-centered approach is increasingly standard in the evaluation and management of valvular heart disease [4].

The purpose of this review is to describe the usefulness of CMR in patients with AS before and after percutaneous intervention, its major limitations, and the future perspectives of this imaging modality in this context, see Table 1. We focus on AS since this is currently the target of most structural aortic valve interventions. Other potential uses, including native aortic regurgitation or prosthetic valve dysfunction [5] fall outside the scope of this review.

## 2. CMR for Aortic Valvular Assessment

Several tests are available to assess the aortic valve prior to intervention, as well as the function of the implanted prosthesis. TTE remains the first-line test because of its wide availability and ease of use [1]. However, there are certain situations, such as poor acoustic windows, inadequate Doppler alignment, acoustic artifacts, or the presence of low flow, which hinder the interpretation of the results and make it necessary to use other modalities [6]. CMR can circumvent these difficulties.

### 2.1. CMR for Aortic Valvular Assessment Prior to TAVR

#### 2.1.1. Aortic Valve Morphology 

**Figure 1:**
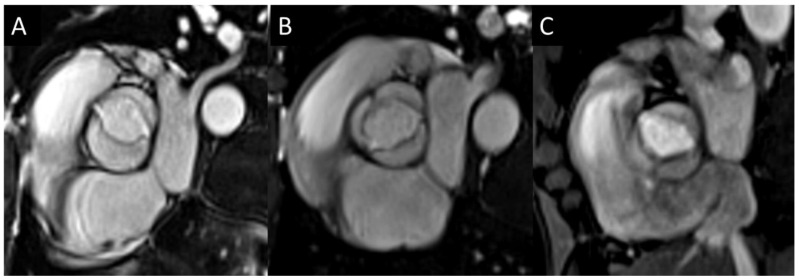
Different types of bicuspid aortic valve (BAV): two-sinus BAV type (**A**); fused BAV type with right-left coronary cusp fusion (**B**); fused BAV with right-non coronary cusps fusion (**C**).

CMR can assess aortic valve morphology with high spatial and temporal resolution and good contrast between blood and valve leaflets. Imagers use balanced steady-state free-precession (SSFP) sequences to obtain short-axis thin-slice views of the valve in motion [7]. With this technique, imagers can assess valve dimensions, the number of leaflets, and, if present, the type of bicuspid valve [8] Figure 1.

#### 2.1.2. AS Quantification 

CMR provides several ways to assess AS severity. The most common approach is aortic valve planimetry. This is based on images of the aortic valve in motion taken throughout the cardiac cycle (cine images). The second is the quantification of blood velocity by velocity-encoded CMR.

##### Aortic Valve Area

Aortic valve planimetry is typically considered the method of choice for grading AS severity. Multiple studies have found that CMR planimetry is highly reliable and reproducible. For example, Kupfahl et al. compared the reliability of aortic valve planimetry between CMR, TTE, transoesophageal echocardiography, and cardiac catheterization in 44 patients. CMR had the best sensitivity and specificity of all non-invasive tests when compared to cardiac catheterization [9].

One limitation of planimetry is a tendency to overestimate the aortic valve area compared to the area obtained by the continuity equation [10]. This is due to the conceptual difference between the anatomical area obtained by planimetry and the functional area estimated by flow evaluation. In addition, the presence of arrhythmias and the relatively low temporal resolution of CMR compared to TTE may reduce the accuracy of identifying the optimal frame during the cardiac cycle with the smallest anatomic aortic valve area [6].

Therefore, planimetry can confirm severe AS when the valve area is less than 1 cm^2^ or exclude severe AS when the valve area is greater than 1.5 cm^2^. However, planimetry should be used cautiously for clinical decision-making when the aortic valve area is between 1 and 1.3 cm^2^ [6].

##### Velocity-Encoded CMR

Two-dimensional (2D) velocity encoding (also known as phase-contrast imaging or flow mapping) is a CMR modality that allows for visualization and quantification of blood velocity. Imagers use the continuity equation to estimate aortic valve area, incorporating blood velocity at the aortic valve and left ventricular (LV) outflow tract, as well as the LV outflow tract area measured on cine images [11]. One of the advantages of CMR over TTE is that, while TTE measures the diameter of the left ventricular outflow tract and assumes that it is circular (it is actually an ellipse), CMR allows for planimetry of the left ventricular outflow tract area without assumptions. Alternatively, pressure gradients can be calculated simply by applying the Bernoulli equation to the velocity obtained through the stenotic aortic valve [12].

Multiple studies have found that velocity-encoded CMR is highly reliable for assessing AS. One early study included 24 patients who underwent both TTE and velocity-encoded CMR. For both imaging techniques, the pressure gradients were calculated using the Bernoulli equation, and the aortic valve area was estimated with the continuity equation. The pressure gradients and valve area obtained by CMR correlated well with the accepted echocardiographic parameters [12].

The main limitation of velocity-encoded CMR is a tendency to underestimate the maximum velocity. The presence of arrhythmias and the relatively low temporal resolution of CMR can reduce the accuracy of identifying the frame during the cardiac cycle that contains the maximum velocity of the AS. In addition, if the sampled velocity is distal or non-orthogonal to the point of maximum velocity, the maximum velocity may be underestimated. Therefore, imagers can consider a peak velocity higher than 4 m/s by velocity-encoded CMR as highly specific for severe AS. On the other hand, if the velocity obtained is lower than 4 m/s, severe AS may not be fully excluded [6]. 

Although velocity-encoded CMR has been shown to be reliable in the assessment of AS, it is relegated to a second step in the evaluation of AS when accurate aortic valve planimetry cannot be calculated.

In recent years, a technique has been developed to partially overcome these limitations. Four-dimensional (4D) phase-contrast imaging allows for blood velocity to be measured in three spatial dimensions throughout the cardiac cycle, eliminating the need for the imaging plane to be perpendicular to the stenotic jet and making it easier to identify the point of highest velocity. A recent study included 18 patients with suspected severe AS based on TTE. All patients underwent 4D flow imaging prior to aortic valve intervention, and eight of them underwent invasive measurement of AS severity. The peak pressure gradient obtained with 4D flow was comparable to that obtained invasively. In addition, it had a better association with the 6 min walk test and post-intervention LV mass regression than TTE [13]. The main limitations of 4D phase-contrast imaging are the need for specialized software (not always available), data storage of large data sets, and its long acquisition time (several minutes), which makes it susceptible to respiratory motion [14].

### 2.2. CMR to Guide TAVR

Pre-procedural imaging is critical to guide the selection of the appropriate prosthesis and assess vascular access. Contrast-enhanced CCT is used often because of its three-dimensional renderings, high spatial resolution, and short acquisition time [15]. However, CCT exposes patients to ionizing radiation and requires the use of iodinated contrast, which can worsen renal function in patients with chronic kidney disease. This is an important limitation because a significant number of patients with severe AS suffer from chronic kidney disease [16]. As a result, CMR has been proposed as an alternative imaging modality for pre-TAVR assessment.

Several studies have shown promising results regarding the value of CMR for the assessment of the aortic root and vascular access [17]. One study, published in 2011, compared CMR with CCT for the analysis of aortic root dimensions before TAVR and showed a good correlation (r = 0.86, *p* < 0.001) for aortic root measurements [18].

**Figure 2:**
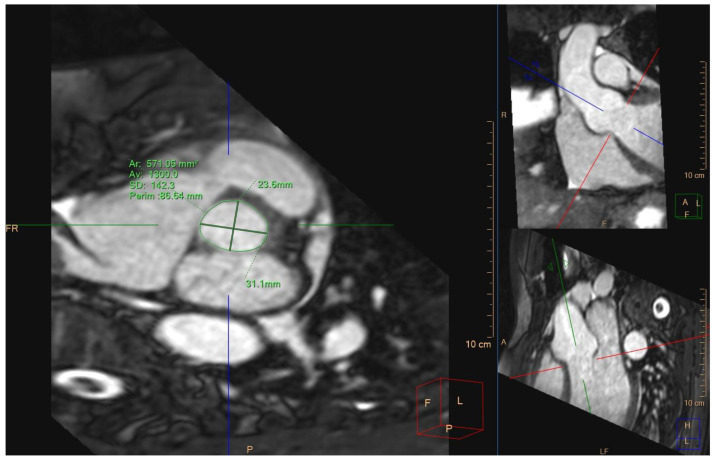
Aortic annulus sizing (maximum diameter, minimum diameter, area, and perimeter) with cardiac magnetic resonance using 3D steady-state free precession imaging by perpendicular reference axes of the analysis software (red, green and blue lines) on two orthogonal views of the aorta; short axis of the aortic annulus (**left panel**); coronal view of the aorta (**top right panel**); sagittal view of the aorta (**bottom right panel**).

In a more recent publication, the authors designed a randomized trial to assess the non-inferiority of CMR to CCT prior to TAVR [19]. The researchers measured the aortic annulus (perimeter, diameters, and area) by CMR using ECG-triggered, non-contrast, free-breathing, 3D SSFP sequences from the left ventricular outflow tract to the ascending aorta, see Figure 2. In addition, the researchers assessed vascular access using 3D gadolinium-enhanced angiography covering the supra-aortic and femoral arteries [19]. The primary outcome was defined as device success at discharge (absence of procedural mortality, correct placement of a single prosthetic valve in the appropriate anatomic location, and proper intended performance of the TAVR). Secondary outcomes included all-cause and six-month mortality, stroke or transient ischemic attack, acute kidney injury, life-threatening bleeding, coronary artery occlusion requiring intervention, major vascular complications, valve-related dysfunction requiring reintervention, and permanent pacemaker implantation. A total of 121 and 127 patients were randomized to undergo CMR or CCT, respectively. The primary outcome was achieved in 92.6% of the CMR group vs. 90.6% of the CCT group (between-group difference of 2% [90% CI, −3.8% to 7.8%]; *p* < 0.01 for non-inferiority). The secondary outcomes were similar between the two groups, except for the incidence of stroke or transient ischemic attack and of permanent pacemaker implantation, which were higher in the CCT-guided group [19]. The average duration of CMR was 35 min, compared to 10 min for CT. However, it should be noted that the protocol used in this study only included sequences for the evaluation of the aortic annulus, aorta, and peripheral vessels. The assessment of cardiac function, cardiac volumes, and myocardium would require additional imaging that would increase the duration of the study. The CMR-guided group was more likely to be treated with a balloon-expandable valve (93% vs. 83% [*p* = 0.01]) compared to patients in the CT group [19].

### 2.3. CMR for Aortic Valve Assessment Following TAVR

**Figure 3:**
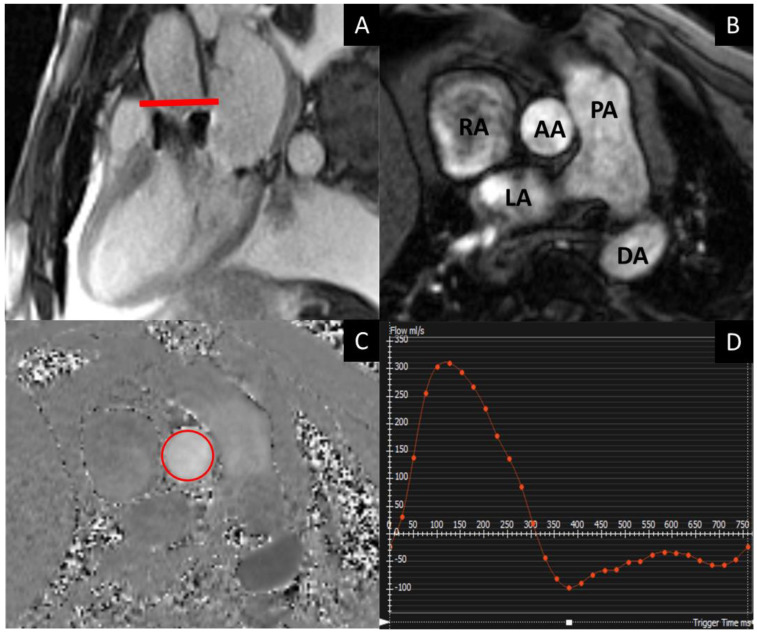
TAVR patient with severe aortic regurgitation. Left ventricular outflow tract view, where the red line indicates where the 2D velocity-encoded measurement is performed (**A**); phase-contrast velocity mapping acquisition showing a magnitude image, where the anatomy can be assessed (**B**); and the velocity map, with aortic contours traced (red circle) (**C**); resulting flow curve from the phase-contrast analysis showing holodiastolic flow reversal consistent with severe aortic regurgitation (**D**). AA: ascending aorta, LA: left atrium, RA: right atrium, DA: descending aorta, PA: pulmonary artery).

The presence of paravalvular regurgitation after TAVR implantation has been associated with a worse prognosis [20]. The most commonly used modality for paravalvular regurgitation assessment is TTE, due to its wide availability and short duration [1]. However, TTE tends to underestimate leak severity and can suffer from technical difficulties, such as a poor acoustic window, limited visualization of eccentric leaks, and artifacts from calcium or the prosthesis itself [21,22]. Similarly, direct evaluation of the valve with CMR is limited due to artifacts related to strut metal in the prosthesis. In contrast, 2D velocity-encoded CMR, with the imaging plane placed in the ascending aorta just beyond the TAVR, provides superior accuracy and reproducibility for quantifying leak severity [23], see Figure 3.

One study quantified aortic regurgitation and calculated the regurgitant fraction and volume in 90 patients after TAVR using velocity-encoded CMR and 3D TTE. The results were compared with the grading of aortic regurgitation using 2D TTE-based Valve Academic Research Consortium (VARC)-2 criteria. CMR grading of aortic regurgitation did not agree with 2D TTE-based VARC-2 criteria in a significant number of patients. The intra- and interobserver variabilities in the assessment of regurgitant volume and fraction after TAVR were substantial with 2D TTE, lower with 3D TTE, and very low with CMR [24].

Another study included 135 patients in whom aortic regurgitation was quantified using velocity-encoded CMR and Doppler TTE after TAVR. Over 26 months of follow-up, a higher regurgitant fraction after TAVR was associated with increased mortality (hazard ratio: 1.18 for each 5% increase in regurgitant fraction [95% confidence interval: 1.08 to 1.30]; *p* < 0.001) and the combined endpoint of mortality and rehospitalization for heart failure (hazard ratio: 1.19 for each 5% increase in regurgitant fraction; 95% confidence interval: 1.15 to 1.23; *p* < 0.001). Regurgitant fraction by CMR had a stronger association with mortality and rehospitalization for heart failure after TAVR compared to the grading of aortic regurgitation by TTE. A regurgitant fraction ≥30% was the best predictor of worse clinical outcomes [25].

The 4D flow CMR has also been used to assess the severity of paravalvular regurgitation after TAVR. A recent study evaluated the usefulness of TTE, standard 2D flow mapping (gold standard), and 4D flow CMR for assessing paravalvular regurgitation after TAVR in 21 patients [26]. On TTE, no paravalvular regurgitation was classified as more than mild, whereas 2D and 4D flow mapping classified three (15.8%) as moderate or severe. Additionally, the regurgitant fraction measured by standard 2D and 4D flow mapping showed a good correlation. However, it is unclear which technique, CMR or TTE, was more accurate.

## 3. CMR for Left Ventricular Assessment

### 3.1. AS-Related Changes in Left Ventricular Adaptation

The LV remodels are in response to the chronic pressure overload of severe AS. At an early stage, myocyte hypertrophy is accompanied by increased collagen production by myofibroblasts, resulting in diffuse interstitial fibrosis. Eventually, myocytes may be replaced by fibrotic tissue (myocyte cell death), resulting in focal replacement fibrosis [27,28]. CMR is well-suited for the evaluation of all these processes.

### 3.2. CMR for Left Ventricular Assessment in AS

Different tests are available to evaluate the myocardium. Endomyocardial biopsy remains the gold standard, but it is an invasive test with associated risks [29]. In addition, this test has lower sensitivity, since samples are obtained from a small region of the right ventricular myocardium and may miss pathological tissue. CCT provides LV dimensions and some myocardial characterization [30] but is not as well-validated as CMR. TTE can assess LV dimensions and parameters that may be associated with myocardial fibrosis (e.g., systolic and diastolic dysfunction) but does not offer any direct form of myocardial characterization.

CMR is considered the gold standard for the noninvasive measurement of LV mass, volumes, and remodeling. The presence of arrhythmias and the relatively low temporal resolution of CMR compared to TTE may reduce the accuracy of identifying the optimal frame during the cardiac cycle with the true end-diastolic and end-systolic volumes [6].

Also, CMR can characterize and quantify myocardial abnormalities such as fibrosis [31]. Focal (replacement) fibrosis can be detected by late gadolinium enhancement (LGE) and diffuse interstitial (reactive) fibrosis can be assessed with parametric imaging [27,28].

LGE has become a crucial tool for assessing the myocardium in both ischemic and non-ischemic cardiomyopathies. The term LGE refers to the transient accumulation of gadolinium-based contrast agents in areas of myocardial scar a few minutes after administration. The presence and distribution of LGE help identify the etiology of the underlying process (e.g., coronary artery disease, sarcoidosis, amyloidosis, myocarditis, etc.), as well as inform long-term prognosis [32,33]. Identification of ischemic-type LGE could potentially be helpful in determining the need (or lack thereof) for further evaluation of underlying coronary disease, such as pre-TAVR invasive angiography, although this application has not been validated. 

CMR parametric imaging provides quantitative maps in which the signal intensity of each image voxel is proportional to a specific tissue property. One of these techniques is T1 mapping (evaluated pre- and post-contrast administration), which provides information on T1 time, a magnetic property that depends on the characteristics of that tissue (in this case the myocardium) [34]. Two types of parametric imaging are commonly used in clinical practice to assess diffuse myocardial fibrosis. Pre-contrast T1 mapping measures native myocardial T1, which provides information on its composition, including the intracellular and extracellular spaces. Native T1 mapping is dependent also on imaging parameters such as the strength of the magnetic field. However, the combination of pre-contrast and post-contrast T1 measurements of the myocardium and the blood, accounting for the hematocrit, allows for the quantification of the extracellular volume (ECV), a parameter that is relatively independent of imaging technique or contrast dose. ECV represents the extracellular space (where the gadolinium contrast is deposited) and is typically expressed as a percentage of the myocardium [27]. Native T1 and ECV are increased with any disease that increases extracellular space (i.e., fibrotic tissue, amyloid, or edema). Native T1 mapping and ECV have been validated in different clinical scenarios including for patients with AS, with good correlation with the presence of myocardial fibrosis on intraoperative biopsy [35,36]. The combination of parametric imaging and LGE patterns can be used to diagnose relatively common coexisting conditions, such as cardiac amyloidosis, which is more prevalent in low-flow low-gradient AS, although further validation is needed [37].

#### 3.2.1. CMR for Ventricular Assessment Prior to TAVR

##### LV Remodeling

CMR can characterize several patterns of LV remodeling in AS that have prognostic value independent of the stenosis severity [6]. Kwiecinski et al. evaluated the prognostic impact of asymmetric wall thickening in a prospective observational cohort study of 166 patients with AS. They found that asymmetric wall thickening was associated with biomarkers of myocardial injury and predicted aortic valve replacement or death independent of stenosis severity [33]. TTE was less sensitive than CMR, missing one-third of cases of asymmetric wall thickening.

##### Myocardial Fibrosis

**Figure 4:**
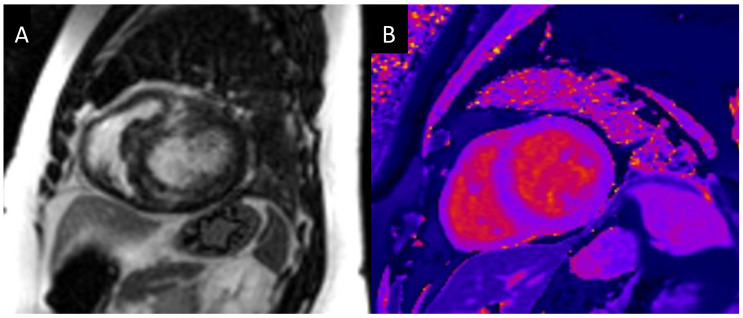
Focal myocardial fibrosis assessed by late gadolinium enhancement (**A**). Diffuse myocardial fibrosis assessed by precontrast T1-time (1150 ms) (**B**).

Multiple studies have shown that LGE is a frequent finding in patients with moderate or severe AS (Figure 4A). Barone-Rochette et al. showed that up to 28% of patients with severe AS had LGE. Among these, 32% had an ischemic pattern and 68% a non-ischemic pattern. Within the group with non-ischemic LGE, 68% had focal enhancement, 21% had a diffuse pattern, and 11% had septal linear LGE [28]. Another study, which included 143 consecutive patients with moderate–severe AS, showed that up to 94 patients (66%) had LGE. There was a typical pattern of prior myocardial infarction in 40 patients (28%), midwall LGE in 54 patients (38%), and a dual pattern in 8 patients (6%). In this study, there was no difference between the severity of AS and the presence or absence of LGE [38]. Furthermore, LGE has also been shown in patients with low-flow low-gradient AS. In this particular group, a higher burden of fibrosis by biopsy and of LGE compared to high-gradient AS was observed in one study [39], although it was not confirmed in a different investigation [40]. 

The presence of LGE in AS has prognostic implications. The largest study to date evaluated 674 patients with severe AS before aortic valve surgery and showed LGE in 51% of patients (18% infarct pattern and 33% non-ischemic pattern). Three hundred ninety-nine patients underwent valve replacement and 275 underwent TAVR. Patients were followed for a mean of 3.6 years, during which 21.5% died. In a multivariable analysis, the presence of a scar was independently associated with all-cause mortality (HR, 2.39; 95% CI, 1.40–4.05; *p* = 0.001) [41]. These findings were confirmed in a pooled meta-analysis that included 19 studies and 2032 patients with AS. LGE was present in 49.6% of patients, 63.6% of whom showed a non-ischemic type. The presence of LGE was associated with higher all-cause mortality (OR [95% CI] = 3.26 [1.72, 6.18], *p* = 0.0003) and cardiovascular mortality (OR [95% CI] = 2.89 [1.90, 4.38], *p* < 0.0001) [42]. 

Pre-contrast T1 time and ECV (Figure 4B) have also been shown to be independent predictors of adverse events in AS [43,44]. One study prospectively enrolled 127 patients with moderate or severe AS and 33 controls. Myocardial native T1 time was longer in patients with AS than in the controls (1232 ± 53 ms vs. 1185 ± 37 ms; *p* = 0.008). The majority of events, including death, occurred in the highest T1 tertile group [43]. A second study investigated the prognostic value of ECV for patients with severe AS. The study included 440 patients who underwent CMR two weeks prior to aortic valve surgery. After a median follow-up of 3.8 years, ECV was associated with mortality (HR: 1.15; 95% CI: 1.07 to 1.23; *p* < 0.001) and cardiovascular death (Hazard ratio: 1.22; 95% CI: 1.07 to 1.38; *p* < 0.003) in the univariable analysis [44]. 

#### 3.2.2. CMR for Ventricular Assessment after TAVR

##### LV Reverse Remodeling after TAVR

CMR can be used to monitor improvements in LV dimensions and function after valve TAVR. An early CMR study showed improvement in cardiac function, volume, and mass after TAVR implantation [45]. These findings were more recently confirmed in another investigation in which CMR was performed in 40 patients with AS before and 1 year after valve replacement. There was an improvement in the LV ejection fraction, LV global longitudinal and circumferential strain, left atrial strain, and total myocardial mass, as well as recovery from heart failure [46]. 

##### Myocardial Fibrosis after TAVR

CMR also can be used to monitor myocardial fibrosis after aortic valve replacement.

In one study, 58 patients with severe symptomatic AS underwent CMR and endomyocardial biopsy prior to surgical aortic valve replacement. CMR was repeated 9 months after surgery and showed no change in the degree of LGE [47]. Similarly, Treibel et al. found no changes in LGE after predominantly surgical, but also transcatheter, valve replacement, suggesting that replacement fibrosis (scar) does not regress [48]. Furthermore, new areas of LGE, presumably of embolic etiology, may develop after TAVR [49].

On the other hand, interstitial fibrosis appears to be, at least in part, reversible. Hwang et al. studied 43 patients with severe AS who underwent aortic valve replacement [50]. Native T1 was assessed by CMR 1 month before surgery and 1 year after surgery. There was a significant reduction in native T1 on follow-up, which was associated with a reduction in LV mass. Importantly, there were fewer adverse clinical events at follow-up in the group of patients who normalized native T1 after surgery. 

Regarding ECV changes after intervention, there are conflicting findings reported in the literature. While, for instance, the latter study found no overall changes in ECV, with reductions in patients with post-surgical T1 normalization, others have found counterintuitive increases in ECV after valve replacement [48,51]. However, this is due to the fact that ECV reflects the degree of interstitial space expansion as a percentage of total myocardial mass, which also decreases with AS relief. To overcome this problem, investigators developed the indexed ECV, which reflects the total volume of LV extracellular space in absolute rather than relative terms. [6]. In the aforementioned study by Treibel et al., ECV increased, but both indexed ECV (or matrix volume) and cell volume decreased [48]. However, the reduction in cell volume was larger than that of the matrix volume (22% and 16%, respectively), which is consistent with a faster regression of LV myocyte hypertrophy than of diffuse fibrosis and results in a paradoxical increase in ECV [48]. Importantly, these changes were accompanied by improvements in functional class, diastolic function, and natriuretic peptides. Similar findings have been also reported after TAVR [46].

## 4. Potential Limitations of CMR

In addition to the specific limitations of CMR in grading the severity of AS, guiding TAVR (discussed above), and assessing the myocardium with LGE, CMR has other general limitations that should be mentioned.

CMR requires patient cooperation with the ability to lie down, remain still, and, ideally, follow breathing instructions. Images need to be synchronized with the heart, which requires adequate triggering from physiologic signals, such as the ECG or peripheral pulse, and the presence of arrhythmias may degrade the image quality and limit the accuracy of quantitative measures [52].

On the other hand, the use of gadolinium contrast has its limitations. It requires intravenous access and, in patients with advanced chronic kidney disease, has been associated with the development of nephrogenic systemic fibrosis. Nevertheless, although it should be used with caution in patients with a glomerular filtration rate <30 mL/min per 1.73 m^2^, current agents typically do not constitute a formal contraindication if otherwise clinically indicated [53].

In addition, CMR is costly, and imaging protocols can be long and complex, sometimes taking up to 60 min per patient [54]. This, together with the factors discussed previously, may influence availability and explain the heterogeneity observed in the performance of CMR depending on the type of country and the expertise of the center where CMR is performed [55]. 

## 5. Conclusions and Future Perspectives

CMR is a versatile imaging modality that allows for a complete evaluation of the patient with severe AS. It can assess AS severity accurately and provide myocardial characterization. Similar to CT, the most widely used modality for TAVR planning, CMR allows for the assessment of annular dimensions, vascular access, and cardiac function and volume in a single examination, although without the need for radiation or the use of iodinated contrast. However, acquisition times are long, availability is lower, and extensive experience is required for both acquisition and interpretation. In addition, there are intrinsic limitations, such as the evaluation of valve calcification or mechanisms of post-TAVR leaks, and more supporting evidence is still needed in several areas such as the selection of valve type.

Efforts are being directed toward technological advances to reduce acquisition times and make this imaging modality more accessible. These include “real-time” sequences that acquire information at a higher speed, eliminating respiratory and cardiac motion artifacts, and “all-in-one” approaches for simultaneous multiparametric evaluation (e.g., cine and tissue characterization) [54]. These advances can make imaging more efficient and help design “TAVR-specific” protocols. Advances in artificial intelligence may also facilitate data analysis and the identification of new prognostic factors [56]. At the same time, clinical trials are underway to investigate the prognostic role of tissue characterization for determining the optimal timing of surgery in asymptomatic patients with severe AS. (NCT03094143). The results of these studies may facilitate the expansion of CMR in the evaluation of patients with severe AS prior to structural aortic valve procedures.

## Figures and Tables

**Table 1:** Applicability of various imaging modalities for percutaneous aortic valve interventions.

	Echocardiography	Cardiac CT	Cardiac MRI
**Pre-TAVR assessment**			
Assessment of severity of aortic stenosis	+++	+	++
Leaflet count and morphology	++	++	++
Aortic valve calcification	++	+++	+
Sizing of aortic root, annulus, and LVOT	++	+++	+++
Location of coronary ostia	+	+++	++
Ventricular volumes and function	++	++	+++
Myocardial characterization (e.g., fibrosis)	+	+	+++
Vascular access		+++	++
Extracardiac findings	+	+++	++
**Post-TAVR assessment**			
Severity of paravalvular leak	++	+	+++
Hypoattenuated leaflet thickening	+	+++	
Myocardial reverse remodeling	++	+	+++
**Use in selected populations**			
Renal insufficiency	+++	+	++
Minimal radiation preferred	+++		+++
**Feasibility**			
Availability	+++	++	+
Portability	+++		
Cost	+	++	+++
